# Targeting the KAT7/H3K14ac/RAC2 Axis Mediates OXPHOS to Promote Stemness Maintenance and Malignancy in Glioblastoma

**DOI:** 10.1002/advs.77551

**Published:** 2026-09-08

**Authors:** Jilong Liu, Yanfei Sun, Yuehua Zhu, Guangjing Mu, Jiazheng Wang, Liangliang Wang, Feihu Zhao, Zhimin Zhao, Jian Wang, Ying He, Zheng Jiang, Xingang Li, Mingzhi Han, Bin Huang

**Affiliations:** ^1^ Department of Neurosurgery Qilu Hospital Cheeloo College of Medicine and Institute of Brain and Brain‐Inspired Science Shandong University Jinan China; ^2^ Jinan Microecological Biomedicine Shandong Laboratory Shandong Key Laboratory of Brain Health and Function Remodeling Jinan China; ^3^ Department of Neurosurgery The Second Qilu Hospital of Shandong University Cheeloo College of Medicine Shandong University Jinan China; ^4^ Department of Biomedicine University of Bergen Bergen Norway; ^5^ Research Center for Basic Medical Sciences Qilu Hospital of Shandong University Jinan China

**Keywords:** GBM, GSCs, KAT7, OXPHOS, RAC2

## Abstract

Glioblastoma multiforme (GBM), the most lethal type of primary brain tumor, exhibits profound metabolic plasticity driven by glioma stem cells (GSCs), which sustain therapeutic resistance and tumor recurrence. Here, we elucidate a novel epigenetic‐metabolic axis mediated by the histone acetyltransferase KAT7 that orchestrates oxidative phosphorylation (OXPHOS) dominance in GSCs. Through a multi‐omics analysis, we demonstrated that KAT7 is preferentially upregulated in GBM, particularly in the classical subtype and in GSC‐enriched populations, where it activates Rac family samll GTPase 2 (RAC2) expression via H3K14 acetylation of its promoter. Mechanistically, KAT7‐mediated RAC2 upregulation triggers PAK1/2/3 phosphorylation, increasing tricarboxylic acid cycle (TCA) and ATP production. Genetic ablation of KAT7 impairs GSCs self‐renewal, induces apoptosis, and suppresses tumor growth in orthotopic xenograft models. Conversely, KAT7 overexpression or pharmacological activation of the KAT7–RAC2 axis restores metabolic fitness and malignant phenotypes. Notably, the small‐molecule inhibitor WM‐3835, which targets KAT7, exhibits potent anti‐GBM efficacy by disrupting H3K14ac and mitochondrial respiration, leading to prolonged survival in mice. Our study identifies KAT7 as a master regulator of GSCs metabolism, revealing an actionable therapeutic target in GBM progression. Targeting the KAT7–RAC2–PAK axis may represent a precise strategy to overcome metabolic plasticity‐driven therapeutic resistance in this recalcitrant malignancy.

## Graphical abstract

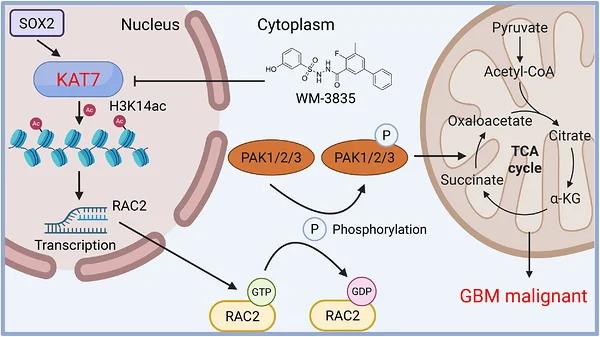

## 1 Introduction

As the most aggressive primary malignant brain tumor, glioblastoma multiforme (GBM) accounts for 45%–50% of gliomas [1, 2], and is characterized by a short survival period and limited therapeutic efficacy. The malignant progression and therapeutic resistance of glioblastoma (GBM) arise largely from its complex heterogeneous landscape, which manifests at both the intertumoral level and intratumoral level [3], wherein glioma stem cells serve as the central protagonists [4, 5]. GSCs are a unique subpopulation of tumor cells endowed with a self‐renewal capacity and multilineage differentiation potential. In addition to initiating tumor growth, GSCs can also evade conventional treatments by maintaining stemness traits and driving tumor recurrence under therapeutic pressure [6]. Targeting GSCs is pivotal for informing the development of innovative pharmacological strategies against GBM.

Unlike other cellular subtypes in tumor samples, GSCs exhibit metabolic plasticity distinct from that of differentiated tumor cells, characterized by their dynamic ability to switch between glycolysis and oxidative phosphorylation (OXPHOS) in response to microenvironmental changes and therapeutic pressures. GSCs possess a distinct mitochondrial metabolic architecture, marked by an enriched content and increased activity of electron transport chain components. This configuration enables robust ATP synthesis through OXPHOS, allowing functional adaptation to hypoxic and nutrient‐variable microenvironments [7, 8, 9]. This metabolic adaptability not only sustains the ATP supply and mitochondrial function in GSCs but also preserves their tumorigenic potential during metabolic stress. Targeting these metabolic features of GSCs may provide novel strategies for GBM therapy, but limited treatments are available for clinical use.

As defining hallmarks of cancer, epigenetic and metabolic reprogramming exhibit GBM malignancy‐regulating effects and mediate communication at the molecular level [4, 10, 11]. The dynamic process of histone acetylation serves as a key regulator of the chromatin architecture and gene accessibility. Catalyzed by histone acetyltransferases (HATs), acetylation of lysine residues relaxes condensed chromatin (heterochromatin) into an open conformation (euchromatin), facilitating transcription [12, 13, 14]. This effect is reversed by histone deacetylases (HDACs), ensuring precise control of metabolic gene expression profiles. Therefore, a reciprocal relationship exists between acetyl‐CoA availability and histone acetylation. Signal‐dependent changes in acetyl‐CoA concentrations dynamically recalibrate the histone acetylation landscape, thereby influencing transcription. Conversely, histone acetylation acts as a homeostatic mechanism, releasing acetyl moieties to help sustain cytosolic acetyl‐CoA pools under fluctuating energy conditions [15, 16]. Driven by the goal of developing novel therapeutics, significant research efforts are focused on discovering potent and selective small molecules that target specific HATs and other enzymes, leading to the progression of several agents into clinical trials [17].

The histone acetyltransferase KAT7 (HBO1/MYST2), a core MYST family member, specifically acetylates histones H3K14 and H4 at K5, K8, and K12. Crucially, KAT7 is responsible for the genome‐wide presence of H3K14ac, a modification vital for transcriptional activation [18]. The enzymatic activity and substrate specificity of KAT7, which lacks intrinsic chromatin‐binding domains, are conferred by its association with specific scaffold proteins. Complexes containing BRPF proteins direct H3 acetylation (e.g., H3K14ac), whereas JADE‐associated complexes target H4 (H4K5/8/12ac) and are essential for the initiation of DNA replication [19, 20, 21]. The oncogenic functions of KAT7 are context‐dependent. It supports leukemogenesis by acetylating H3K14 on stemness gene promoters, increases glycolysis in head and neck squamous cell carcinoma (HNSCC) by modifying lactate dehydrogenase A (LDHA), and promotes colorectal carcinogenesis through the H3K14ac–MRAS–MAPK/ERK axis [22, 23, 24]. However, its role and molecular mechanisms in GBM have not yet been defined.

In this study, we aimed to define a novel KAT7–RAC2–PAK signaling axis that drives OXPHOS dominance in glioblastoma stem cells (GSCs). Elucidating the mechanism by which KAT7 remodels the GSCs' epigenome to sustain OXPHOS dominance contributes to the identification of actionable targets and ideas for novel therapeutic agents for GBM.

## 2 Results

### 2.1 The OXPHOS‐Related Protein KAT7 is Highly Expressed in Gliomas and Leads to a Poor Prognosis

**FIGURE 1:**
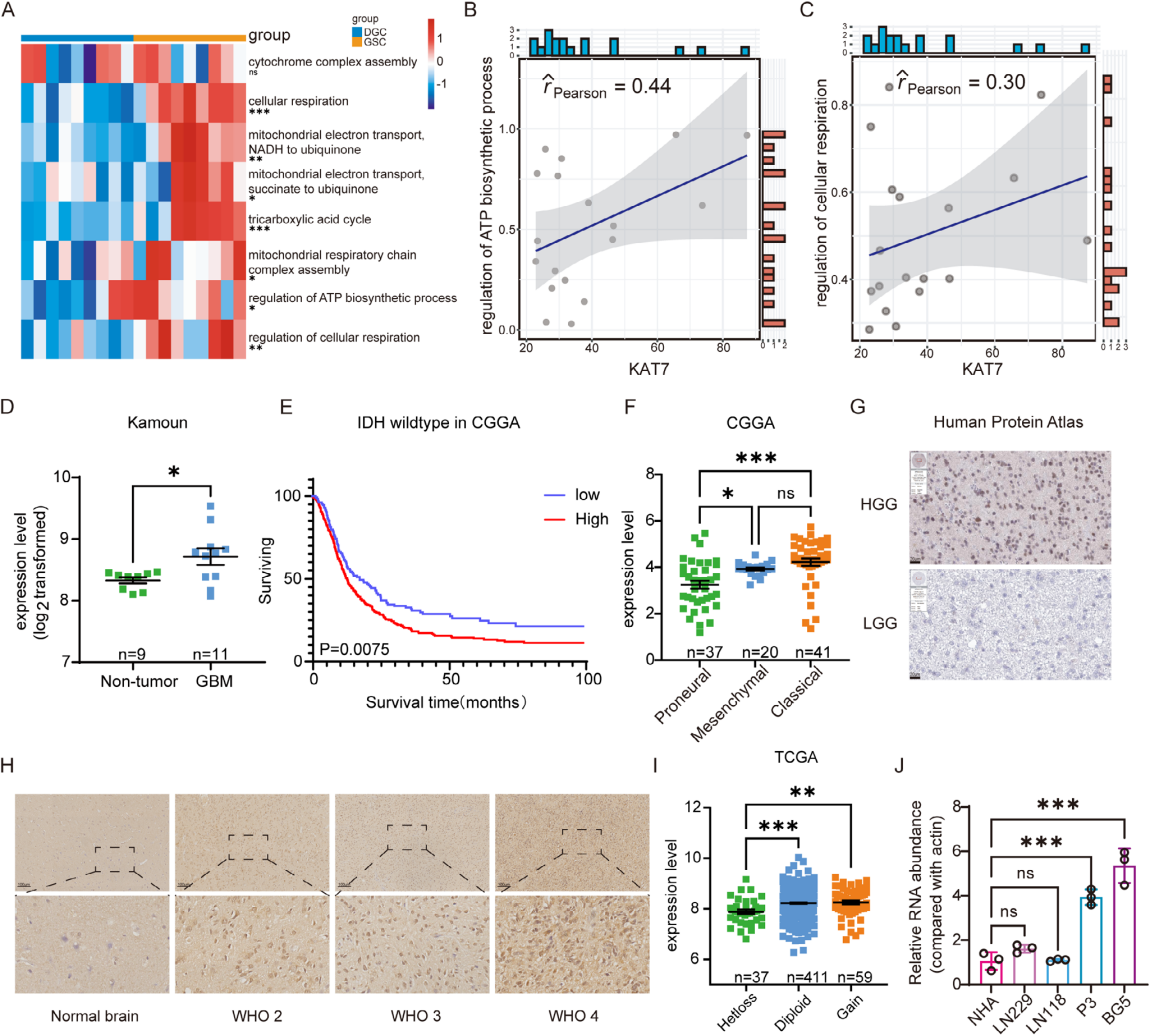
KAT7 is highly expressed in gliomas and leads to a poor prognosis. (A) Single‐sample GSEA of metabolic gene sets in 3 GSCs and matched differentiated GBM cells (DGCs) from the HumanCyc database. The data are shown as the means ± the standard errors of the means (SEMs. ^***^ *p* < 0.001;^**^ *p* < 0.01; and ^*^ *p* < 0.05. Statistical significance was determined with an unpaired *t* test. (B, C) Correlations between KAT7 expression and metabolism‐related biological process scores in the GSE54791 dataset. Statistical significance was determined with the *F* test. (D) Expression of KAT7 in GBM and nontumor samples in the Kamoun database. *n* indicates the number of biologically independent samples. The data are shown as the means ± the standard errors of the means (SEMs). ^*^ *p* < 0.05. Statistical significance was determined with a two‐sided Student's *t* test. (E) Kaplan–Meier plots of IDH wild‐type samples in the CGGA database based on high or low KAT7 expression in tumors (*n* = 295 in the KAT7 high group; *n* = 129 in the KAT7 low group). A log‐rank test was used to assess statistical significance. (F) KAT7 expression in 3 GBM subtypes from the CGGA dataset. *n* indicates the number of biologically independent samples. The data are shown as the means ± the standard errors of the means (SEMs). ^***^ *p* < 0.001 and ^*^ *p* < 0.05. Statistical significance was determined with one‐way ANOVA. (G) Representative images of IHC staining for KAT7 in HGG and LGG tissues from the Human Protein Atlas database. Scale bar = 20 µm. (H) Representative images of IHC staining for KAT7 in normal brain tissues and gliomas of different grades. (*n* = 3). Scale bar = 100 µm. (I) KAT7 expression in tissues with three types of gene copy number variations from TCGA datasets. The data are shown as the means ± the standard errors of the means (SEMs). ^***^ *p* < 0.001 and ^**^ *p* < 0.01. Statistical significance was determined with one‐way ANOVA. (J) qRT‒PCR analysis of KAT7 mRNA expression in normal human astrocytes and different glioma cells. ACTB was used as an internal control. The data are shown as the means ± the standard errors of the means (SEMs). ^***^ *p* < 0.001. Statistical significance was determined using one‐way ANOVA.

We investigated the candidate mitochondrial respiration‐related proteins involved in GSCs by selecting a subset of cellular respiration‐ and mitochondrial metabolism‐related pathways from the HumanCyc database and integrating them with the GSE54791 dataset to analyze metabolic alterations in glioma stem cells [25]. Our findings revealed increased cellular respiration and upregulated mitochondrial metabolism in GSCs compared with differentiated GSCs (Figure 1A Tables S1 and S2). These findings suggest that glioma stem cells require an increased energy supply to maintain their stemness properties and malignant phenotype.

Previous studies have shown that KAT7 plays a crucial role in neural stem cell plasticity, indicating its potential role in stemness manipulation [26, 27]. Moreover, we observed that the expression level of KAT7 in the GSE54791 dataset was significantly correlated with metabolic pathways highly expressed in GSCs (Figure 1B,C). We analyzed KAT7 expression levels in the Kamoun datasets to further determine the function of KAT7 in glioma progression and observed that KAT7 expression was significantly upregulated in gliomas compared with normal brain tissue (Figure 1D). In addition, high scores were also associated with a poor prognosis in IDH wild‐type samples according to the CGGA database (Figure 1E). The pan‐cancer analysis of the KAT7 gene conducted in the GEPIA2 database also revealed its high expression in GBM (Figure S1A). Further analysis revealed higher KAT7 expression in the classical and mesenchymal molecular subtypes of GBM (Figure 1F). These results demonstrated that increased expression of KAT7 was associated with the malignant progression of glioma [28, 29]. Based on data from the Human Protein Atlas database, we observed that KAT7 was highly expressed state in high‐grade gliomas (HGGs) (Figure 1G). We performed IHC staining for KAT7 in an independent cohort comprising 9 glioma samples and 3 control brain tissue samples to verify this conclusion. KAT7 protein expression was significantly increased in GBM compared with both LGG and normal brain tissues (Figure 1H). In the TCGA‐GBM database, KAT7 expression was strongly positively correlated with the copy number variation status, with a stepwise trend: the lowest expression was observed in the heterozygous loss group, intermediate expression was observed in the diploid group, and the highest expression was observed in the copy number gain group (Figure 1I) [30]. Furthermore, qRT‒PCR analysis revealed that compared with human astrocytes (NHAs), primary GBM cells (GBM#P3 and GBM#BG5) exhibited significantly higher KAT7 expression, suggesting a potential role for KAT7 upregulation in the maintenance of GSCs and the progression of GBM (Figure 1J). These results collectively underscore that the expression of the OXPHOS‐related protein KAT7 is a candidate prognostic biomarker for GBM, suggesting its potential to predict patient outcomes.

### 2.2 **KAT7** is Expressed at Higher Levels in GSCs

**FIGURE 2:**
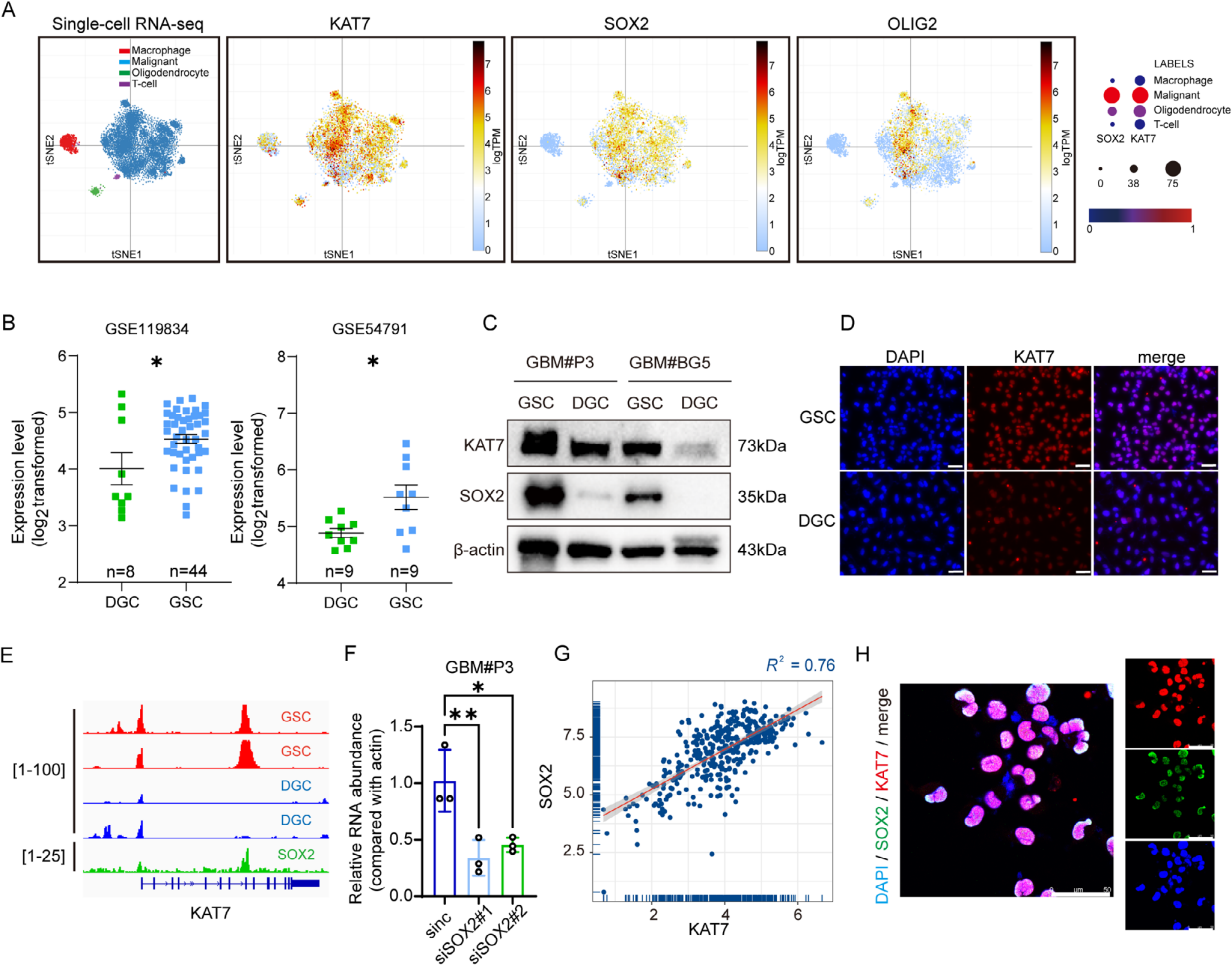
KAT7 is relatively highly expressed in glioma stem cells. (A) Single‐cell RNA‐seq analysis of differential KAT7, SOX2, and OLIG2 expression in GBM. (B) KAT7 expression in DGCs and GSCs in the GSE119834 and GSE54791 datasets. n indicates the number of biologically independent samples. The data are shown as the means ± the standard errors of the means (SEMs). ^*^ *p* < 0.05. Statistical significance was determined with a two‐sided Student's *t* test. (C) Western blot analysis of KAT7 expression in GBM#P3 and GBM#BG5 cells and differentiated cells. (D) Fluorescence intensity of KAT7 in GSCs and DGCs within GBM#P3 cells. Scale bar = 100 µm. (E) Genome browser view of H3K27ac and SOX2 levels on the KAT7 gene body in GSCs and DGCs. (F) qRT‒PCR analysis of KAT7 expression in GBM#P3‐shControl, ‐shSOX2#1 and ‐shSOX2#2 cells. ACTB was used as an internal control. The data are presented as the means and SEMs (*n* = 3). ^**^ *p* < 0.01 and ^*^ *p* < 0.05. Statistical significance was determined with one‐way ANOVA. (G) Correlations between KAT7 expression and the SOX2 score based on the CGGA database. Statistical significance was determined with the *F* test. (H) IF staining for KAT7, SOX2, and DAPI in GBM#P3 cells. Scale bar = 50 µm.

We initially evaluated the coexpression of KAT7 and SOX2 in malignant subpopulations through an analysis of single‐cell data to further validate the relationship between KAT7 and the maintenance of glioma stemness (Figure 2A). An analysis of the GSE119834 and GSE54791 datasets revealed a consistent and statistically significant upregulation of the KAT7 mRNA in glioma stem cells compared with that in differentiated glioma cells (DGCs), suggesting its potential role in GSCs maintenance and stemness properties (Figure 2B) [25, 31]. We induced the differentiation of primary glioma cells (GBM#P3 and GBM#BG5) by supplementing the primary culture medium with fetal bovine serum (FBS), which is a well‐established method to trigger the differentiation of stem‐like tumor cells [32], to further confirm these findings. Western blot analysis revealed that KAT7 expression was significantly lower in differentiated cells than in undifferentiated control cells, providing direct evidence for the involvement of KAT7 in maintaining the stem‐like phenotype of glioma cells (Figure 2C). Moreover, compared with that in GSCs, the fluorescence intensity of KAT7 in DGCs was significantly reduced (Figure 2D and Figure S1B). Previous studies have demonstrated that SOX2 plays a pivotal role in gliomas, functioning as a core transcription factor essential for maintaining the stemness characteristics of glioma stem cells and serving as a critical driver of gliomagenesis, therapeutic resistance, and tumor recurrence [33]. From an epigenetic perspective, SOX2 is closely associated with H3K27ac modification peaks, where it can modulate H3K27ac states to facilitate glioma progression [34]. We observed a significant increase in the H3K27ac peak intensity within the KAT7 gene region in GSCs compared with that in DGCs, along with a colocalization of SOX2 enrichment sites at KAT7 loci, revealing the epigenetic regulation of KAT7 expression by H3K27ac and SOX2 (Figure 2E). For further confirmation, we performed qRT‒PCR and found that SOX2 knockdown in GBM#P3 cells significantly suppressed KAT7 levels (Figure 2F). Simultaneously, we performed a correlation analysis between KAT7 and SOX2 expression levels in the CGGA database and found a significant positive correlation (R^2^ = 0.76), indicating that higher KAT7 expression is consistently associated with elevated SOX2 expression in glioma tissues (Figure 2G). Immunofluorescence staining also revealed that KAT7 and SOX2 were colocalized in the nucleus, with KAT7 demonstrating both chromatin‐associated and cytoplasmic localization patterns (Figure 2H and Figure S1C).

Collectively, these findings indicate that KAT7 is highly expressed in glioma stem cells, where it serves as a critical regulator of stemness maintenance, with its expression likely regulated by SOX2‐mediated transcriptional mechanisms.

### 2.3 KAT7 Knockdown Inhibits GBM Growth and the Maintenance of GSCs Both In Vitro and In Vivo

**FIGURE 3:**
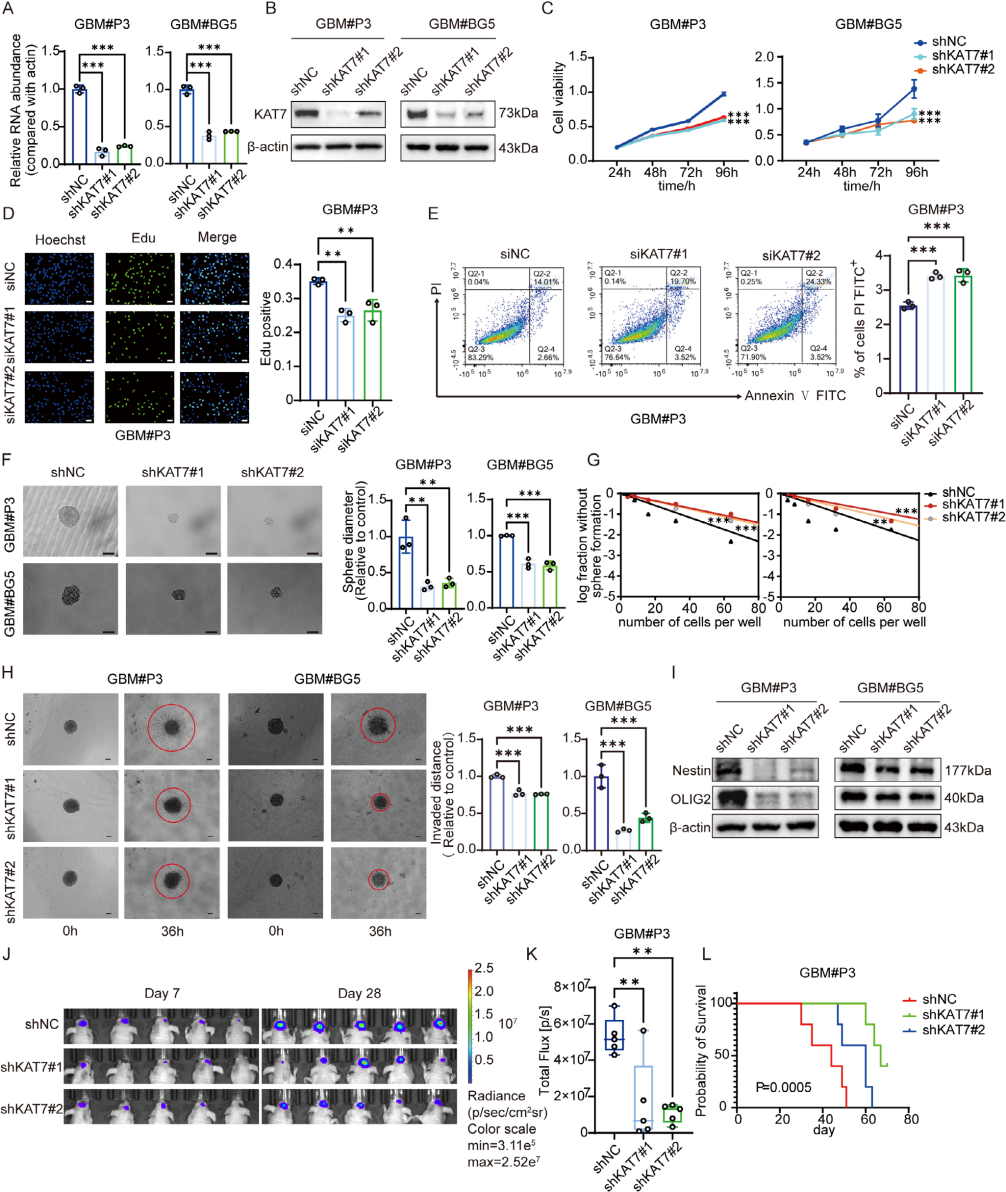
KAT7 knockdown inhibits GBM growth and GSCs maintenance in vitro and in vivo. (A) qRT‒PCR analysis of KAT7 expression in GBM#P3‐ and GBM#BG5‐shNC, ‐shKAT7#1 and ‐shKAT7#2 cells. *ACTB* was used as an internal control. The data are presented as the means and SEMs (*n* = 3). ^***^ *p* < 0.001. Statistical significance was determined with one‐way ANOVA. (B) Western blot analysis of KAT7 expression in GBM#P3 and GBM#BG5‐shNC, ‐shKAT7#1, and ‐shKAT7#2 cells. (C) CCK‐8 assay to detect cellular activity after knockdown of KAT7 in GBM#P3 and GBM#BG5‐shNC, ‐shKAT7#1, and ‐shKAT7#2 cells. The data are presented as the means and SEMs (*n* = 3). ^***^ *p* < 0.001. Statistical significance was determined with 2‐way ANOVA. (D) EdU staining of GBM#P3‐siNC, ‐siKAT7#1, and ‐siKAT7#2 cells. Scale bar = 100 µm. The data are presented as the means and SEMs (*n = 3*). ^**^ *p* < 0.01. Statistical significance was determined with one‐way ANOVA. (E) Flow cytometry analysis of apoptosis in GBM#P3‐siNC, ‐siKAT7#1, and ‐siKAT7#2 cells detected by Annexin V‐FITC and PI staining. The means and SEMs are shown (*n = 3*). ^***^ *p* < 0.001. Statistical significance was determined with one‐way ANOVA. (F) Representative images of tumor sphere formation assays using GBM#P3 and GBM#BG5‐shNC, ‐shKAT7#1, and ‐shKAT7#2 cells. Scale bar = 100 µm. The means and SEMs are shown (*n* = 3). ^***^ *p* < 0.001 and ^**^ *p* < 0.01. Statistical significance was determined with one‐way ANOVA. (G) Extreme limiting dilution assays for GBM#P3 and GBM#BG5‐shNC, ‐shKAT7#1, and ‐shKAT7#2 cells. The data are presented as the means ± the SEMs of 3 independent experiments. ^***^ *p* < 0.001 and ^**^ *p* < 0.01. Statistical significance was determined with pairwise tests. (H) Three‐dimensional tumor spheroid invasion assay to assess the invasion of GBM#P3 and GBM#BG5‐shNC, ‐shKAT7#1, and ‐shKAT7#2 cells. Scale bar = 100 µm. The means and SEMs are shown (*n* = 3). ^***^ *p* < 0.001. Statistical significance was determined with one‐way ANOVA. (I) Western blot analysis of Nestin and Olig2 expression in GBM#P3 and GBM#BG5‐shNC, ‐shKAT7#1, and ‐shKAT7#2 cells. (J, K) Bioluminescence images and the corresponding quantification of tumors in mice implanted with GBM#P3‐shNC, ‐shKAT7#1, and ‐shKAT7#2 cells at week 4. The means and SEMs are shown (*n* = 5). ^**^ *p* < 0.01. Statistical significance was determined with one‐way ANOVA. (L) Kaplan–Meier survival curves of tumor‐bearing mice implanted with GBM#P3‐shNC, ‐shKAT7#1, or ‐shKAT7#2 cells (*n* = 5 mice per group). A log‐rank test was used to assess statistical significance.

We depleted KAT7 in glioblastoma stem cells to investigate its specific function. By establishing stable knockdown models in GBM#P3 and GBM#BG5 cell lines with lentivirus‐delivered KAT7‐targeting shRNAs (shControl, shKAT7#1, and shKAT7#2), we conducted a series of in vitro experiments to assess its effects. qPCR and Western blot analyses confirmed the significant downregulation of KAT7 expression (Figure 3A,B). CCK8 and EdU assays demonstrated that the inhibition of KAT7 decreased cell viability and inhibited cancer cell proliferation (Figure 3C,D). An apoptosis assay showed that the inhibition of KAT7 expression induced apoptosis (Figure 3E). We also found that silencing KAT7 expression attenuated the sphere‐forming ability and self‐renewal ability of GSCs (Figure 3F,G). Furthermore, compared with control spheres, P3‐ and BG5‐KAT7‐SH‐treated spheres exhibited decreased invasion in the 3D spheroid invasion assay (Figure 3H). The downregulation of KAT7 expression also resulted in a significant reduction in the expression levels of the stemness markers Olig2 and Nestin [35, 36], which is consistent with the altered self‐renewal of KAT7‐depleted cells (Figure 3I).

We generated luciferase‐tagged GBM#P3 cells with stable KAT7 knockdown using lentiviral vectors, followed by intracranial implantation into the brains of 4‐week‐old athymic nude mice (*n* = 5) to establish orthotopic xenograft models and investigate the effect of KAT7 depletion on glioblastoma progression in vivo. At 4 weeks post‐implantation, tumor growth was significantly lower in the GBM#P3‐shKAT7 cohort than in the control cohort (Figure 3J,K). Overall survival was also significantly prolonged in P3 tumor‐bearing mice inoculated with sh‐KAT7 than in control mice (Figure 3L). IHC staining revealed that the expression of the proliferation marker Ki‐67 and the stemness marker Olig2 significantly decreased after KAT7 knockdown (Figure S1D–F).

Taken together, KAT7 inhibition suppressed glioma proliferation and the self‐renewal properties of glioma stem cells both in vitro and in vivo.

### 2.4 KAT7 Regulates RAC2 Expression by Catalyzing the H3K14ac Modification

**FIGURE 4:**
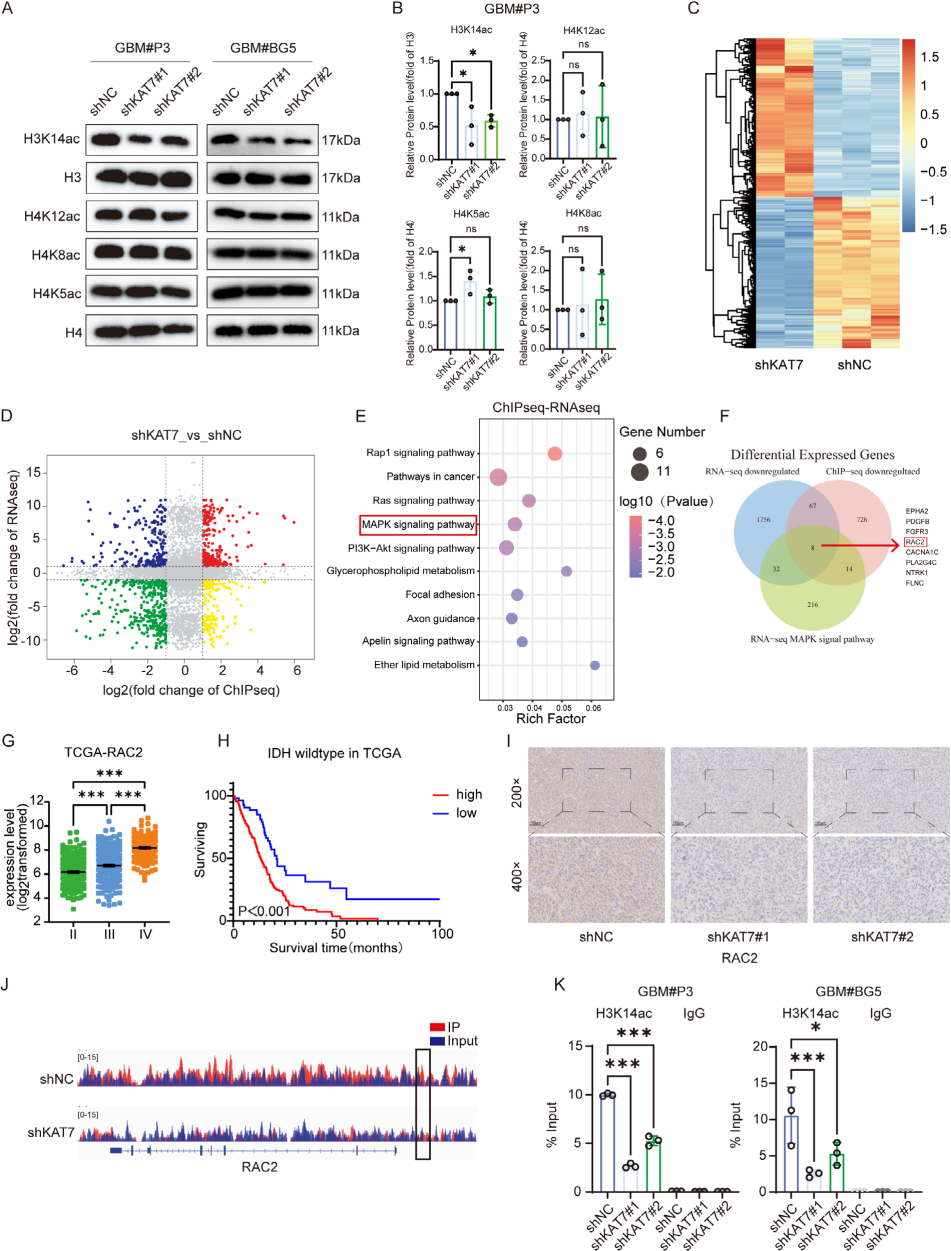
**KAT**7 regulates RAC2 expression by catalyzing the acetylation of histone H3K14. (A, B) Western blot analysisof H3K14ac, H4K12ac, H4K8ac and H4K5ac levels in GBM#P3‐ and GBM#BG5‐shNC, ‐shKAT7#1, and ‐shKAT7#2 cells and corresponding quatification in GBM#P3. The means and SEMs are shown (*n = 3*). ^*^ *p* < 0.05. Statistical significance was determined with one‐way ANOVA. (C) Heatmap of differentially expressed transcripts identified in RNA‐seq data from GBM#P3 cells infected with shNC or shKAT7‐1 lentivirus. The mean gene expression data were *z*‐transformed for display and are colored red for high expression and blue for low expression. (D) Four‐quadrant plot integrating RNA‐seq and H3K14ac ChIP‐seq data from GBM#P3 cells with shNC or shKAT7‐1 lentiviral infection. (E) KEGG analysis of differentially expressed genes based on RNA‐seq and ChIP‐seq data with *P* values. (F) Venn diagram showing genes that overlap between MAPK signaling pathway components and genes whose expression is downregulated following KAT7 knockdown according to RNA‐seq/ChIP‐seq. (G) Expression of RAC2 in gliomas of different grades in the TCGA database. n indicates the number of biologically independent samples. The data are shown as the means ± the standard errors of the means (SEMs). ^***^ *p* < 0.001. Statistical significance was determined with a two‐sided Student's *t* test. (H) Relationship between RAC2 expression in IDH wild‐type samples and the patient prognosis in the TCGA database. (*n* = 175 patients in the RAC2 high group; *n* = 58 patients in the RAC2 low group). A log‐rank test was used to assess statistical significance. (I) Representative images of IHC staining for RAC2 in tumor tissues from a xenograft model. (*n* = 3). Scale bar = 100 µm. (J) Peak plot illustrating the binding of H3K14ac to the promoter region of the RAC2 gene. (K) qPCR analysis of ChIP assays with the H3K14ac antibody or IgG in GBM#P3‐ and GBM#BG5‐shNC, ‐shKAT7#1, and ‐shKAT7#2. The means and SEMs are shown (*n* = 3). ^***^ *p* < 0.001 and ^*^ *p* < 0.05. Statistical significance was determined with one‐way ANOVA.

Previous research has established that KAT7, a key histone acetyltransferase of the MYST family, regulates diverse cellular processes by mediating the acetylation of specific histone lysine residues, namely, H3K14, H4K12, H4K5, and H4K8, which are critical for chromatin remodeling and transcriptional programs [18, 19, 20, 24]. We delineated how KAT7 functional loss affects histone acetylation dynamics in GSCs, by performing targeted KAT7 depletion and subsequently assessing the levels of these four acetylated histone marks. Western blot analysis revealed a marked decrease in histone H3K14ac levels following KAT7 depletion, corroborating its critical role as a histone acetyltransferase responsible for H3K14 acetylation in GSCs (Figure 4A,B).

We performed ChIP‐seq utilizing an anti‐H3K14ac antibody and RNA‐seq analyses with KAT7 knockdown cells to further determine the role of KAT7‐mediated H3K14ac alterations in glioblastoma malignancy. Comparative transcriptomic profiling revealed 1,863 downregulated genes and 1,960 upregulated genes (*p* < 0.05, |log2FC| > 1) (Figure 4C). We integrated ChIP‐seq and RNA‐seq for comprehensive analysis (Figure 4D). A total of 75 genes were downregulated according to RNA‐seq, with lower levels of the H3K14ac modification. Through an integrated analysis, we discovered that the MAPK signaling pathway was significantly enriched according to the KEGG analysis, and among these genes, the key gene RAC2 exhibited significant decreases in its expression levels and levels of the H3K14ac modification (Figure 4E,F). By analyzing data from TCGA and CGGA databases, we demonstrated that elevated RAC2 expression was significantly correlated with a higher glioma malignancy grade and poor survival outcomes for patients (Figure 4G,H and Figure S1G,H). Moreover, in an orthotopic intracranial xenograft model established with KAT7‐knockdown GBM#P3 cells, IHC staining showed that RAC2 expression was significantly decreased in the knockdown group (Figure 4I). At the same time, we also found that silencing RAC2 expression attenuated the sphere‐forming ability and self‐renewal ability of GSCs (Figure S1I,J). Through the analysis of ChIP‐seq data, we detected a significant reduction in the intensity of the peak for the RAC2 transcriptional start site in the KAT7 knockdown group compared with that in the control group (Figure 4J). Subsequently, ChIP‒qPCR was conducted using cell lysates derived from KAT7‐knockdown cells. Primers were designed specifically to target the H3K14ac‐modified region within the RAC2 transcriptional start site. ChIP‒qPCR studies demonstrated that KAT7 depletion significantly decreased the levels of H3K14ac at the target region, suggesting that KAT7 regulates RAC2 expression by modulating H3K14ac levels at its transcriptional start site (Figure 4K).

Taken together, our results showed that KAT7 facilitates H3K14ac deposition at the RAC2 transcriptional start site, thereby increasing RAC2 promoter activity and further promoting the malignant progression of glioma cells.

### 2.5 KAT7 Increases Mitochondrial Oxidative Phosphorylation in GSCs by Increasing RAC2 Expression

**FIGURE 5:**
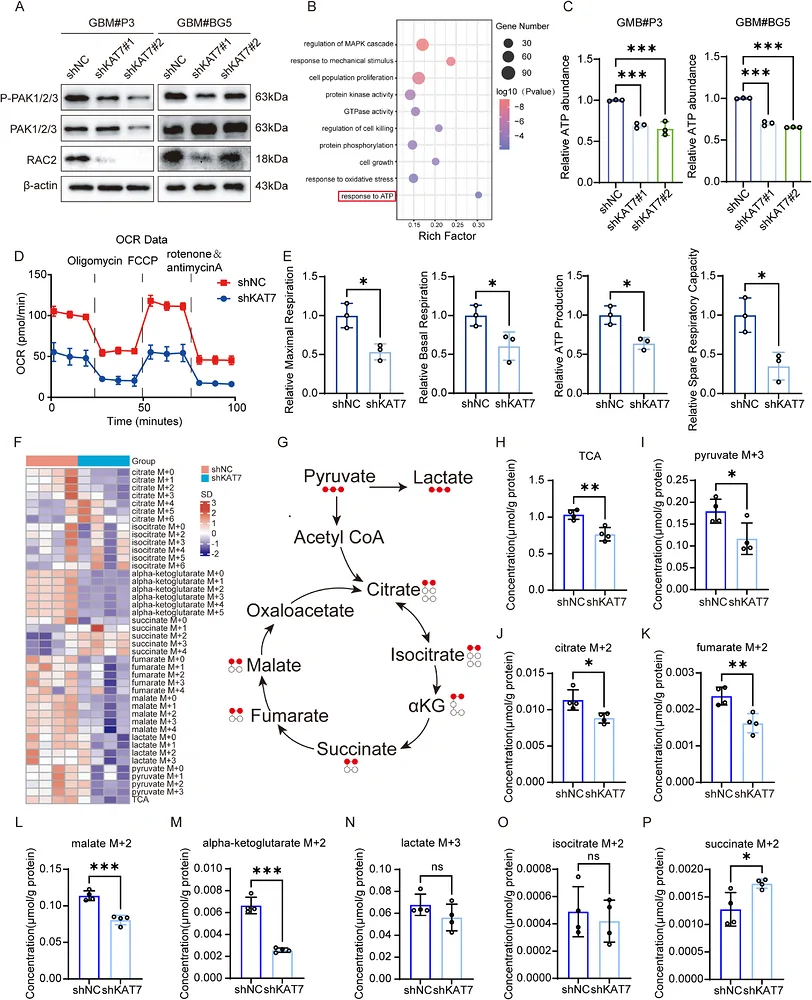
KAT7 increases mitochondrial oxidative phosphorylation in GSCs by increasing RAC2 expression. (A) The expression of proteins downstream of the RAC2 signaling pathway was detected by Western blotting after the knockdown of KAT7 in GBM#P3‐ and GBM#BG5‐shNC, ‐shKAT7#1, and ‐shKAT7#2 cells. (B) GO analysis of differentially expressed genes based on the RNA‐seq analysis, along with *P* values. (C) Representative images of the relative ATP abundance in GBM#P3‐ and GBM#BG5‐shNC, ‐shKAT7#1, and ‐shKAT7#2 cells. The means and SEMs are shown (*n* = 3). ^***^ *p* < 0.001. Statistical significance was determined with one‐way ANOVA. (D) Mitochondrial respiration, indicated as the oxygen consumption rate (OCR), of GBM#P3 cells was measured after consecutive injections of oligomycin (Oligo at 1.5 µm), FCCP (1 µm), and rotenone/antimycin A (0.5 µm). (E) The relative maximal respiration, basal respiration, ATP production, and spare respiratory capacity are presented in bar graphs. The means and SEMs are shown (*n* = 3). ^*^ *p* < 0.05. Statistical significance was determined with an unpaired *t* test. (F) Heatmap showing the overall differences in the levels of all the detected metabolites after normalization. Metabolite concentrations are normalized for display and colored red for high expression and blue for low expression. (G) Schematic representation of [U‐13C] glucose incorporation into tricarboxylic acid (TCA) cycle intermediates demonstrating the metabolic fate of uniformly labeled glucose carbons within central metabolic pathways. (H) Total metabolite concentration in GBM#P3‐shNC and ‐shKAT7#1 cells. The means and SEMs are shown (*n* = 4). ^**^ *p* < 0.01. Statistical significance was determined with an unpaired *t* test. I–P: Comparison of metabolite concentrations between GBM#P3‐shNC and ‐shKAT7#1 cells. The means and SEMs are shown (*n* = 4). ^***^ *p* < 0.001, ^**^ *p* < 0.01 and ^*^ *p* < 0.05. Statistical significance was determined with an unpaired *t* test.

RAC2 is a plasma membrane‐associated small GTPase that undergoes a dynamic cycle between an active GTP‐bound state and an inactive GDP‐bound state [37]. Doye et al. demonstrated that RAC2 regulates the phosphorylation of the p21‐activated kinase family (PAK1/2/3) by modulating its activation dynamics, with this interaction being critical for cytoskeletal remodeling and cell migration pathways [38, 39, 40]. Alterations in PAK1/2/3 activity and phosphorylation status regulate cellular metabolism and energy homeostasis through multiple interconnected pathways, including mitochondrial biogenesis, insulin receptor signaling cascades, fatty acid oxidation, and metabolic reprogramming in cancer cells [41, 42, 43, 44]. Western blot analysis demonstrated that compared with the control treatment, KAT7 depletion significantly suppressed RAC2 protein expression and concomitantly reduced phospho‐PAK1/2/3 levels, suggesting that KAT7 plays an essential role in regulating RAC2–PAK1/2/3 signaling axis activity (Figure 5A). A Gene Ontology (GO) analysis of our RNA‐seq data also revealed that differentially expressed genes were significantly enriched in biological processes related to MAPK signaling pathway regulation and the response to ATP, highlighting their potential roles in tumor aggressiveness and metabolic reprogramming (Figure 5B).

Quantitative ATP measurements preliminarily revealed a significant reduction in ATP levels in the KAT7 knockdown groups compared with those in the control groups, suggesting impaired mitochondrial energy metabolism and the potential disruption of cellular bioenergetics (Figure 5C). Mitochondrial respiratory function was systematically characterized using a Seahorse XF Analyzer with an XF Mito Stress Test Kit in GBM#P3 and GBM#BG5 to further elucidate the function of KAT7 in metabolic reprogramming in GSCs. Sequential pharmacological modulation was performed with 1.5 µm oligomycin (ATP synthase inhibitor, Complex V), 1 µm FCCP (carbonyl cyanide 4‐(trifluoromethoxy)phenylhydrazone, protonophore), and 0.5 µm rotenone/antimycin A (Complex I and III inhibitors, respectively) to establish a concentration‐dependent inhibition profile (Figure 5D and Figure S1K). Compared with the baseline value, the administration of the protonophore FCCP induced an increase in the oxygen consumption rate (OCR), demonstrating a preserved mitochondrial reserve capacity through enhanced electron transport chain (ETC) activity under maximal proton gradient dissipation conditions. Our results revealed that compared with the vehicle treatment, KAT7 inhibition induced a reduction in the maximal mitochondrial respiratory capacity. The mitochondrial basal oxygen consumption rate was also reduced in shKAT7‐expressing GSCs. In addition, compared with those in the control group, ATP production‐coupled respiration rates and spare respiratory capacity were significantly reduced in GBM#P3 and GBM#BG5 after KAT7 inhibition (Figure 5E and Figure S1L).

We further investigated the effect of KAT7 knockdown on the metabolic profile of GBM#P3 cells by performing D‐glucose‐13C_6_ stable isotope tracing combined with a targeted metabolomics analysis. A heatmap of the metabolites clearly revealed a global downregulation pattern in the KAT7 knockdown group, suggesting extensive metabolic reprogramming (Figure 5F). Comprehensive metabolic profiling revealed that compared with control cells, KAT7 knockdown cells presented significantly reduced total concentrations of key metabolites, including pyruvate, lactate, citrate, isocitrate, succinate, fumarate, and malate (Figure 5G,H). This pattern indicates a profound disruption in central carbon metabolism, as these metabolites serve as critical nodes linking glycolysis, the tricarboxylic acid (TCA) cycle, and other biosynthetic pathways. The isotopomer analysis provided deeper insights into the dynamics of metabolic flux. We observed marked decreases in the levels of several labeled glucose‐derived metabolites, such as pyruvate M+3, citrate M+2, fumarate M+2, malate M+2, and alpha‐ketoglutarate (αKG) M+2 (Figure 5I–M). These findings are particularly significant because citrate and malate are essential intermediates in the TCA cycle, whereas pyruvate acts as the primary gateway for glucose‐derived carbons to enter mitochondrial metabolism [45, 46, 47]. The concurrent decreases in the levels of these metabolites underscores a specific impairment in glucose carbon flux through the TCA cycle. In contrast, the levels of lactate M+3 and isocitrate M+2 did not differ significantly between the groups, indicating that KAT7 knockdown does not uniformly affect all metabolic pathways (Figure 5N,O). Notably, succinate M+2 levels were paradoxically increased in the knockdown cells (Figure 5P). This unexpected increase may reflect a compensatory response to energy stress, as succinate can accumulate during mitochondrial dysfunction and potentially serve as an alternative energy substrate or signaling molecule. Collectively, these metabolic tracing data demonstrate that KAT7 depletion leads to three major metabolic alterations in GBM#P3 cells: (1) a global reduction in the levels of central carbon metabolites, (2) specific decreases in the glucose‐derived labeling of pyruvate and TCA cycle intermediates, and (3) potential rerouting of metabolic flux at the succinate node.

These changes strongly suggest that KAT7 plays a critical role in maintaining mitochondrial metabolic activity in GSCs, particularly in facilitating the forward progression of carbon atoms through the TCA cycle.

### 2.6 KAT7 Promotes OXPHOS in GBM Cells via RAC2

**FIGURE 6:**
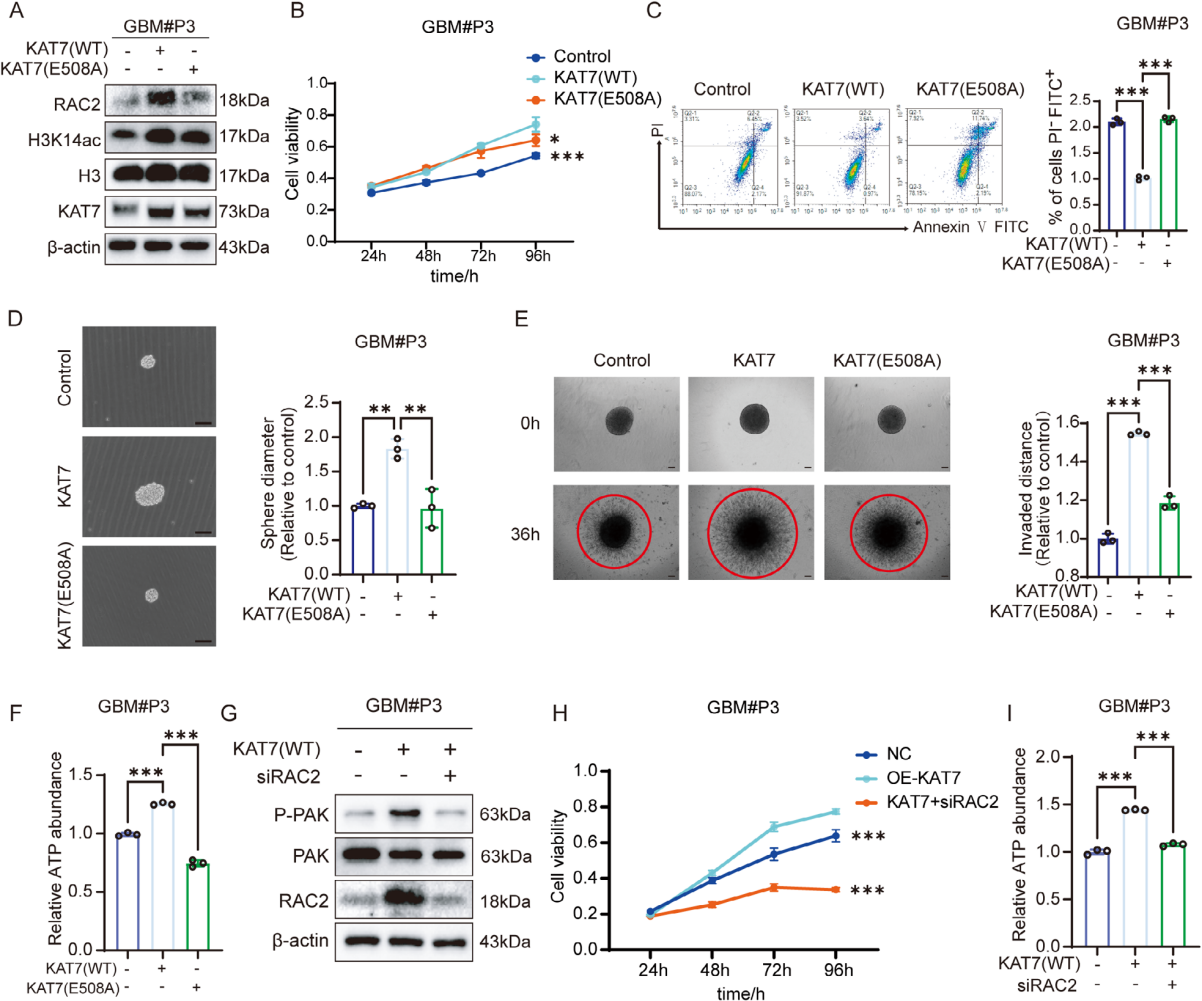
KAT7 promotes energy metabolism in glioma via RAC2. (A) Western blot analysis of KAT7, H3K14ac and RAC2 levels in GBM#P3‐NC, ‐KAT7‐OE and ‐KAT7 (E508A) cells. (B) CCK‐8 assay to detect cellular activity in GBM#P3‐NC, ‐KAT7‐OE and ‐KAT7 (E508A) cells. The means and SEMs are shown (*n* = 3). ^***^ *p* < 0.001 and ^*^ *p* < 0.05. Statistical significance was determined with 2‐way ANOVA. (C) Flow cytometry was used to detect Annexin V‐FITC and PI staining and assess apoptosis in GBM#P3‐NC, ‐KAT7‐OE and ‐KAT7 (E508A) cells. The means and SEMs are shown (*n* = 3). ^***^ *p* < 0.001. Statistical significance was determined with one‐way ANOVA. (D) Representative images of tumor sphere formation assays using GBM#P3‐NC, ‐KAT7‐OE and ‐KAT7 (E508A) cells. Scale bar = 100 µm. The means and SEMs are shown (*n* = 3). ^**^ *p* < 0.01. Statistical significance was determined with one‐way ANOVA. (E) Three‐dimensional tumor spheroid invasion assay to assess the invasion of GBM#P3‐NC, ‐KAT7‐OE and ‐KAT7 (E508A) cells. Scale bar = 100 µm. The means and SEMs are shown (*n* = 3). ^***^ *p* < 0.001. Statistical significance was determined with one‐way ANOVA. (F) Representative images of the relative ATP abundance in GBM#P3‐NC, ‐KAT7‐OE and ‐KAT7 (E508A) cells. The means and SEMs are shown (*n* = 3). ^***^ *p* < 0.001. Statistical significance was determined with one‐way ANOVA. (G) The expression of proteins downstream of the RAC2 signaling pathway was detected by Western blotting after the overexpression of KAT7 and knockdown of RAC2 in GBM#P3 cells. (H) CCK‐8 assay to detect cellular activity in GBM#P3 cells after overexpression of KAT7 and knockdown of RAC2. The means and SEMs are shown (*n* = 3). ^***^ *p* < 0.001. Statistical significance was determined with 2‐way ANOVA. (I) Representative images of relative ATP abundance in GBM#P3 cells after the overexpression of KAT7 and knockdown of RAC2. The means and SEMs are shown (*n* = 3). ^***^ *p* < 0.001. Statistical significance was determined with one‐way ANOVA.

We established a lentivirus‐mediated overexpression system in GBM#P3 cells to determine the roles of KAT7 in GBM progression and the RAC2 regulatory networks. Wild‐type KAT7 and the catalytic mutant KAT7 E508A were cloned and inserted into a lentiviral vector with puromycin resistance. Western blot experiments showed that compared with the control group, the groups with both wild‐type KAT7 overexpression and mutant KAT7 overexpression presented significantly increased KAT7 protein levels (Figure 6A). Notably, KAT7 overexpression increased H3K14ac levels in cells expressing the wild‐type protein but not mutant KAT7, indicating that the mutation disrupted the ability of KAT7 to regulate H3K14ac. Consistent with the alterations in wild‐type KAT7 and mutant KAT7 expression, the protein levels of RAC2 also exhibited parallel changes. Compared with the control group and the mutant KAT7‐overexpressing group, the wild‐type KAT7‐overexpressing group exhibited significantly enhanced cell proliferation, as shown by a CCK8 assay, and reduced apoptosis, as indicated by apoptosis assays (Figure 6B,C). Moreover, KAT7 overexpression significantly enhanced the sphere‐forming ability and invasive capacity of GBM#P3 cells (Figure 6D,E). Quantitative ATP measurements also revealed a significant increase in ATP levels in the KAT7‐overexpressing groups (Figure 6F). These findings further confirm the critical role of KAT7 in promoting malignant progression and maintaining stemness in glioma.

We knocked down RAC2 expression in GBM#P3 OE‐KAT7 cells to further validate the role of the KAT7/RAC2 axis in glioma growth. The Western blot results showed that RAC2 knockdown inhibited the increased phosphorylation of PAK1/2/3 induced by KAT7 overexpression (Figure 6G). The results of the **CCK**‐8 assay showed that RAC2 knockdown counteracted the increased cell proliferation induced by KAT7 overexpression (Figure 6H). Additionally, RAC2 knockdown suppressed the enhancement of cellular stemness induced by KAT7 overexpression (Figure S1M,N). In addition, through a quantitative ATP test, we showed that the overexpression of wild‐type KAT7 increased cellular energy metabolism, whereas RAC2 knockdown abolished this effect (Figure 6I).

Overall, our findings confirm the tumor‐promoting role of the KAT7–RAC2 signaling axis and suggest a novel therapeutic strategy for GBM.

### 2.7 WM‐3835 Inhibits KAT7 to Suppress Glioma Progression

**FIGURE 7:**
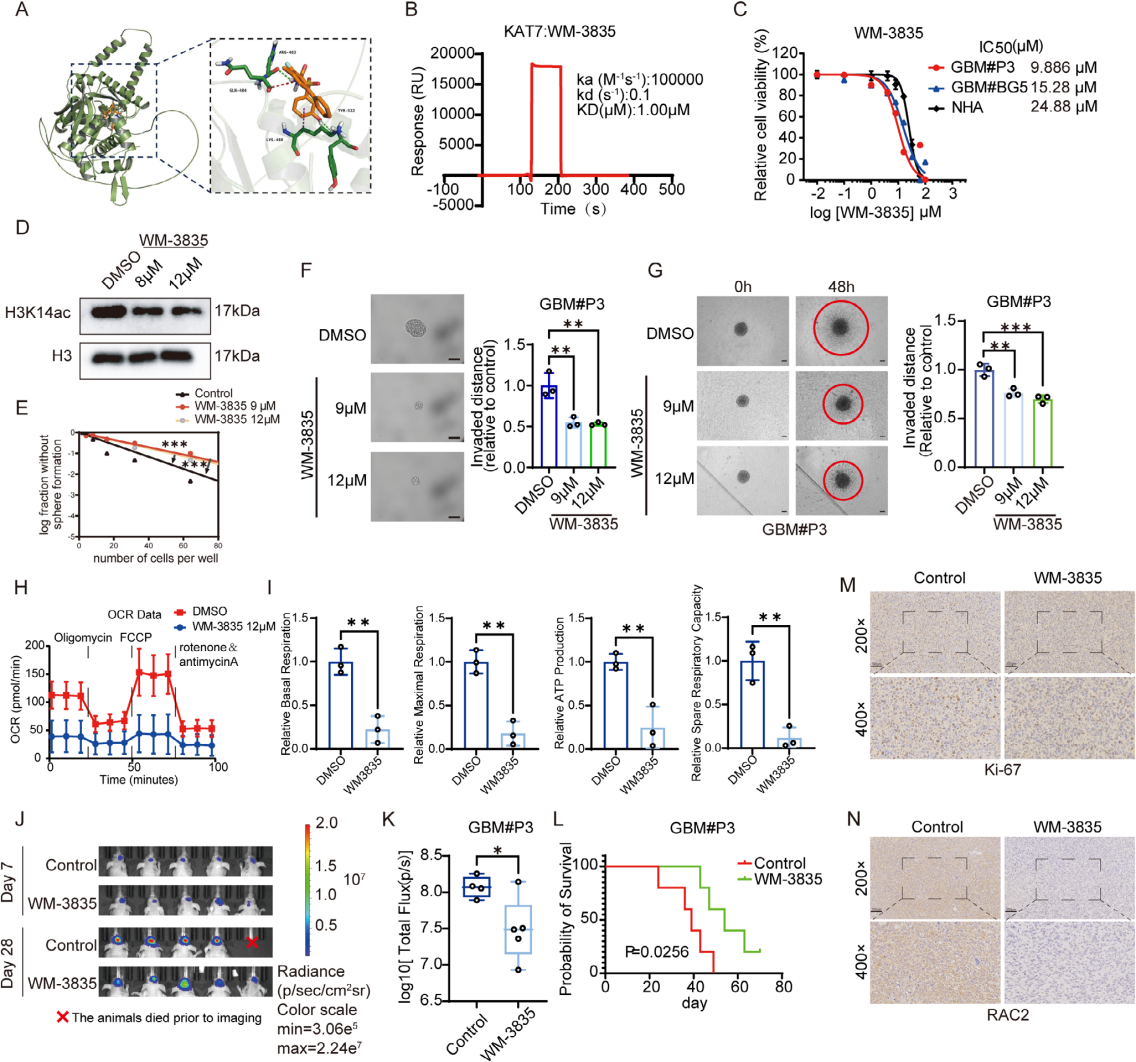
WM‐3835 inhibits KAT7 to suppress glioma progression. (A) Results of molecular docking between KAT7 and WM‐3835. (B) SPR assay showing the global interaction of WM‐3835 with KAT7. (C) CCK‐8 assay for determining the relative viability of NHA, GBM#P3 and GBM#BG5 cells treated with different concentrations of WM‐3835 (*n* = 3) to generate dose–inhibition curves and calculate IC50s. (D) Western blot analysis of the levels of the H3K14ac modification after treatment with different doses of WM‐3835 and DMSO. (E) Extreme limiting dilution assays of GBM#P3 cells treated with WM‐3835. The data are presented as the means ± SEMs of 3 independent experiments. ^***^ *p* < 0.001. Statistical significance was determined with pairwise tests. (F) Representative images of tumor sphere formation assays for GBM#P3 cells treated with WM‐3835. Scale bar = 100 µm. The means and SEMs are shown (*n* = 3). ^**^ *p* < 0.01. Statistical significance was determined with one‐way ANOVA. (G) Three‐dimensional tumor spheroid invasion assay to assess the invasion of GBM#P3 cells treated with WM‐3835. Scale bar = 100 µm. The means and SEMs are shown (*n* = 3). ^***^ *p* < 0.001 and ^**^ *p* < 0.01. Statistical significance was determined with one‐way ANOVA. (H) Mitochondrial respiration, indicated as the oxygen consumption rate (OCR), of GBM#P3 cells treated with WM‐3835 was measured after consecutive injections of oligomycin (Oligo at 1.5 µm), FCCP (1 µm), and rotenone/antimycin A (0.5 µm). (I) The relative maximal respiration, basal respiration, ATP production, and spare respiratory capacity are presented in bar graphs. The means and SEMs are shown (*n* = 3). ^**^ *p* < 0.01. Statistical significance was determined with an unpaired *t* test. (J, K) Bioluminescence images and the corresponding quantification of tumors in mice implanted with GBM#P3 cells and treated with WM‐3835 (*n* = 5) or saline (*n* = 4) at week 4. The means and SEMs are shown. ^*^ *p* < 0.05. Statistical significance was determined with one‐way ANOVA. (L) Kaplan–Meier survival curves of tumor‐bearing mice implanted with GBM#P3 cells and treated with WM‐3835 (*n* = 5 per group). A log‐rank test was used to assess statistical significance. M.N: Representative images of IHC staining for Ki‐67 and RAC2 in tumor tissues from a xenograft model (*n* = 3). Scale bar = 100 µm.

WM‐3835 is a KAT7 inhibitor that potently suppresses cancer cell growth through the targeted inhibition of KAT7 and a subsequent reduction in H3‐H4 acetylation [22, 24, 48]. Molecular docking analyses revealed that WM‐3835 binds to the acetyl‐CoA binding site of KAT7 (Figure 7A). Surface plasmon resonance (SPR) assays further confirmed the direct and specific interaction between WM‐3835 and the KAT7 protein, demonstrating their direct molecular association (Figure 7B). We investigated the function of WM‐3835 in glioblastoma cell lines by first determining its half‐maximal inhibitory concentration (IC50) in normal NHA (24.88 µm), GBM#P3 (9.886 µm), and GBM#BG5 (15.28 µm) cells (Figure 7C). Western blot analysis revealed that WM‐3835 suppressed H3K14ac (Figure 7D). Furthermore, WM‐3835 significantly impaired the colony‐forming ability and self‐renewal (Figure 7E,F). We performed 3D invasion assays to assess the effect of WM‐3835 on GBM cell invasion, and the results showed that WM‐3835 markedly reduced the invasiveness of GBM#P3 cells (Figure 7G). WM‐3835 treatment markedly impaired mitochondrial function (basal respiration, maximal respiration, ATP production, and spare respiratory capacity) in GBM#P3 cells, as shown by mitochondrial stress tests, indicating that its antiglioma effect is mediated by suppressing respiration (Figure 7H,I).

The inhibitory effect of WM‐3835 on GBM progression was further validated in an orthotopic xenograft mouse model. Briefly, 4‐week‐old specific pathogen‐free (SPF) nude mice were subjected to a stereotactic intracranial implantation of GBM#P3 cells and randomly divided into control and treatment groups. The treatment group received WM‐3835 via an intravenous tail vein injection at a dose of 5 mg/kg administered three times per week. Longitudinal monitoring revealed that compared with the control treatment, WM‐3835 treatment significantly suppressed tumor growth, as evidenced by the reduced tumor volume measured by bioluminescence imaging (Figure 7J,K). More importantly, compared with the control treatment, WM‐3835 treatment markedly extended the median survival time of tumor‐bearing mice (Figure 7L). Mechanistic investigations demonstrated that WM‐3835 treatment significantly reduced proliferative activity, as shown by a decrease in the number of Ki‐67‐positive cells and a decrease in the level of RAC2 in tumor tissues, as determined by IHC staining (Figure 7M,N).

These findings collectively indicate that WM‐3835 exerts potent anti‐GBM effects through the inhibition of tumor cell proliferation and epigenetic modulation of histone acetylation in vivo.

## 3 Discussion

**FIGURE 8:**
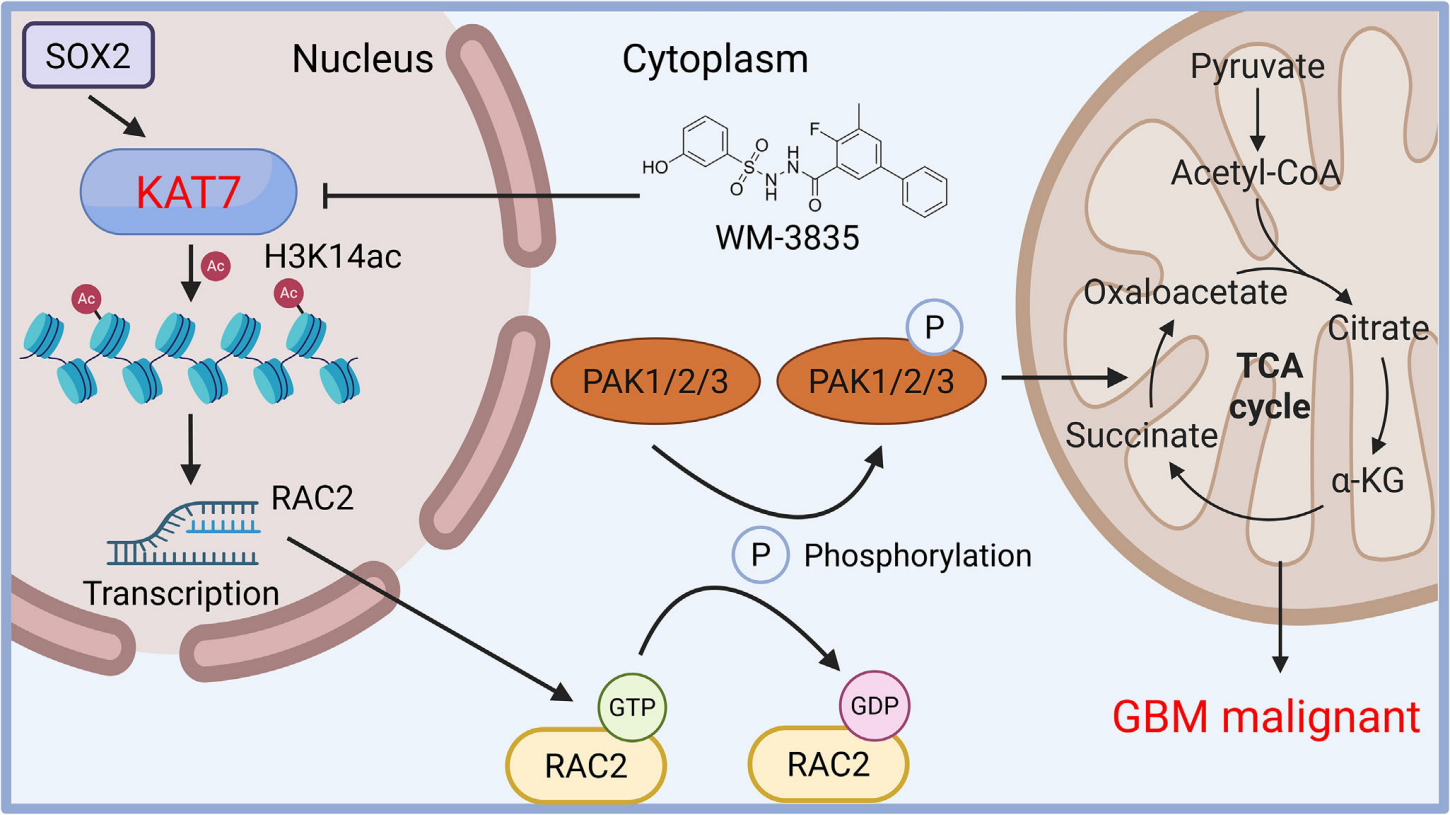
Targeting the KAT7/H3K14ac/RAC2 axis mediates OXPHOS to promote stemness maintenance and malignancy in glioblastoma. SOX2 upregulates KAT7 in glioma stem cells, where KAT7 deposits H3K14 acetylation at the RAC2 promoter to activate RAC2 transcription. RAC2‐GTP subsequently promotes PAK1/2/3 phosphorylation, increasing tricarboxylic acid cycle flux and mitochondrial respiration to support glioblastoma malignancy. Pharmacological inhibition of KAT7 with WM‐3835 disrupts this epigenetic–metabolic axis and restrains tumor progression.

Histone acetylation, a prevalent epigenetic mechanism, has garnered extensive attention because of its critical regulatory role in diverse human malignant tumors, including glioblastoma multiforme [12]. The findings presented in this study provide important insights into the complex interplay between epigenetic modifications and cellular metabolic remodeling in one of the most aggressive human cancers through the identification of KAT7 as a master regulator of tumor progression, stem cell maintenance, and metabolic changes (Figure 8). Our work establishes a novel mechanism by which KAT7‐mediated histone acetylation at the RAC2 transcriptional start site increases its expression, which subsequently activates PAK family protein phosphorylation, thereby reprogramming cellular metabolism and ultimately driving the malignant phenotype of GBM.

Clinical cohort‐based bioinformatics analyses revealed that elevated KAT7 expression in GBM is associated with a gene dosage effect, with a stepwise increase from heterozygous loss to copy number gain. Its preferential overexpression in the classical molecular subtype, which is known for aggressive behavior and therapeutic resistance, particularly underscores its clinical relevance [28, 29]. Immunohistochemical staining of samples from our own cohort also revealed that KAT7 expression progressively increases with the glioma grade. Additionally, the nuclear localization of KAT7 supports its role in chromatin modification and transcriptional regulation. Notably, KAT7 upregulation is more pronounced in primary patient‐derived GBM cells than in normal human astrocytes. The relationship between KAT7 and glioma stem cells represents a particularly important aspect of our findings. The maintenance of stem‐like properties in cancer cells has emerged as a central mechanism of therapeutic resistance and tumor recurrence in GBM [49]. The preferential expression of KAT7 in GSCs demonstrates a robust functional relationship between KAT7 and the preservation of stem cell properties, which is further reinforced by its regulatory interaction with the core stemness factor SOX2. Specifically, the positive correlation between KAT7 and SOX2 expression in clinical samples, coupled with our observation that SOX2 knockdown reduces KAT7 expression, points to functional cooperation between these factors in stemness maintenance.

The inhibition of KAT7 elicits profound antitumor effects on both in vitro and in vivo models of glioblastoma. KAT7 knockdown significantly impaired proliferation, reduced sphere‐forming capacity, and attenuated the invasive potential, concomitant with decreased expression of stemness markers such as Olig2 and Nestin [35, 36], indicating that KAT7 acts both downstream of SOX2 and upstream of stemness maintenance, establishing its essential role in the glioma stem cell regulatory hierarchy. Concurrently, genetic inhibition of KAT7 markedly extended survival in orthotopic xenograft model mice, underscoring its promising therapeutic implications for glioblastoma treatment.

At the molecular level, we revealed that KAT7 exerts its oncogenic effects primarily through the acetylation of histone H3 at lysine 14, a modification that enhances chromatin accessibility and transcriptional activation [19]. RNA‐seq and ChIP‐seq analyses following KAT7 knockdown revealed significant alterations in gene expression profiles, with notable enrichment in pathways related to MAPK signaling and metabolic regulation. Among the differentially expressed genes, RAC2 emerged as a key downstream effector whose expression was directly modulated by KAT7‐mediated H3K14ac. The reduction in H3K14ac levels at the RAC2 transcriptional start site following KAT7 depletion establishes a direct link between KAT7 enzymatic activity and RAC2 expression. These findings are particularly important given the established role of RAC2 in regulating cytoskeletal dynamics and cell migration through its effects on PAK family kinases [38]. Our observation that KAT7 knockdown reduces phospho‐PAK1/2/3 levels provides a plausible mechanism by which the KAT7–RAC2 axis influences multiple aspects of GBM pathophysiology, including invasion, proliferation, and metabolic adaptation. The connection between this pathway and cellular metabolism is further strengthened by our observations that KAT7 depletion leads to reduced ATP levels and impaired mitochondrial function, suggesting that the KAT7–RAC2–PAK axis serves as a critical node integrating epigenetic regulation with bioenergetic processes in GBM cells.

Building upon our observations of cellular bioenergetic alterations, the stable isotope tracing experiments employing D‐glucose‐13C_6_ provide mechanistic insights into how KAT7 inhibition disrupts mitochondrial energy metabolism. Our analyses revealed that KAT7 depletion induces profound changes in central carbon metabolism, with particularly striking impairments in oxidative phosphorylation. The significantly reduced glucose‐derived labeling of pyruvate (M+3), citrate (M+2), fumarate (M+2), and malate (M+2) strongly disrupted the supply of substrates for the electron transport chain, directly compromising the mitochondrial oxidative capacity. While the paradoxical accumulation of succinate (M+2) suggests attempted metabolic compensation through alternative pathways, the overall metabolic signature, characterized by impaired electron flux and reduced ATP coupling efficiency, points to a fundamental breakdown of oxidative phosphorylation. The resulting bioenergetic crisis, marked by depleted ATP pools and a compromised respiratory reserve capacity, ultimately undermines the high energy demands of proliferating GBM cells, providing a metabolic basis for the tumor growth suppression observed in our models.

The successful demonstration of the antitumor activity of WM‐3835 in both in vitro and in vivo models represents a critical translational advance of our study. The specificity of WM‐3835 for KAT7, as evidenced by its binding to the acetyl‐CoA binding site and its reduction of H3K14ac levels, provides a pharmacological tool to validate our genetic findings [22]. The extension of survival in orthotopic xenograft model mice is particularly encouraging, as it suggests that KAT7 inhibition may provide clinical benefit even in the context of established, invasive tumors. The differential sensitivity observed between GBM cells and normal astrocytes, as reflected by their respective IC50 values, suggests a potential therapeutic window that could be exploited in clinical development.

While our work establishes KAT7 as a critical epigenetic regulator in GBM, several limitations warrant discussion. First, our investigation focused primarily on the canonical histone acetyltransferase activity of KAT7, although emerging evidence indicates that KAT7 functions as a multifunctional epigenetic modifier capable of mediating the propionylation and crotonylation of histones [18, 50]. Whether these alternative PTM activities contribute to the oncogenic role of KAT7 in glioma remains unexplored and represents a critical knowledge gap. Second, our mechanistic studies focused on the KAT7–RAC2–PAK axis, yet KAT7 likely regulates additional signaling pathways, given its established role in chromatin accessibility. The comprehensive spectrum of downstream effectors of KAT7 in GBM stem cells requires further study. Third, while the metabolic flux analysis revealed impaired oxidative phosphorylation, the detailed alterations in the mitochondrial and oxidative respiratory chain mediated by KAT7 need further investigation [51]. Finally, although our preclinical models have therapeutic potential, the efficient blood–brain barrier penetration of KAT7 inhibitors remains to be validated, which is essential for clinical translation. These limitations highlight avenues for future research to fully elucidate the complex biological functions of KAT7 in GBM pathogenesis.

In conclusion, our study identifies KAT7 as a pivotal epigenetic regulator in glioblastoma, showing its critical role in coordinating tumor progression through the histone acetylation‐dependent activation of oncogenic signaling pathways and metabolic reprogramming. We elucidated a novel mechanistic axis, KAT7–RAC2–PAK1/2/3, that not only provides deeper insights into the molecular drivers of GBM pathogenesis but also provides a compelling foundation for the development of targeted therapeutic strategies against this aggressive malignancy. Despite the significant progress made, translating these findings into clinical applications remains challenging, particularly in overcoming the inherent therapeutic resistance that has long confounded GBM treatment. Nevertheless, our results provide a promising avenue: epigenetic modulation, particularly through KAT7 inhibition, may hold the key to circumventing this resistance.

## 4 Method

### 4.1 Ethics Statement and Clinical Glioma Tumor Specimens

The research protocol (DWLL‐2024‐053) was approved by the Ethics Committee of Qilu Hospital, Shandong University, and strictly adhered to the relevant regulations and guidelines. The glioblastoma (GBM) tissue samples utilized in this study were obtained from surgical patients at Qilu Hospital, with all participants providing written informed consent. Nontumor brain tissue samples were acquired from patients with severe traumatic brain injury who underwent decompressive surgery, and these patients provided informed consent.

### 4.2 Data Processing and Bioinformatics Analysis

Biological processes related to cellular respiration and mitochondrial metabolism and corresponding terms were retrieved from HumanCyc (https://humancyc.org/). The expression data and associated clinical data were downloaded from GlioVis (http://gliovis.bioinfo.cnio.es/). Transcriptome data for GSCs and DGCs and gene sets related to metabolic processes were obtained from published articles [25, 31]. Images of immunohistochemical (IHC) staining of HGG (https://images.proteinatlas.org/44470/100549_A_4_7.jpg) and LGG (https://images.proteinatlas.org/44470/100549_A_5_1.jpg) were obtained from the Human Protein Atlas (proteinatlas.org). The single‐cell RNA‐seq analysis was based on GSE131928. The results of the pancancer gene expression analysis were obtained from GEPIA2 (http://gepia2.cancer‐pku.cn/#index).

### 4.3 Statistical Analysis

The association between gene expression and mDNAsi was assessed through Pearson's correlation analysis. Two‐group comparisons were conducted using paired or unpaired Student's *t* tests, while multigroup comparisons employed one‐way ANOVA, with all analyses performed using GraphPad Prism software. The survival analysis was performed by constructing Kaplan‒Meier curves and comparing them using the log‐rank test. The data are presented as the means ± SDs/SEMs, with statistical significance set at *p*< 0.05.

### 4.4 Cell Culture

The primary GBM cell lines GBM#P3 and BG5 were derived from patient surgical specimens, characterized, and routinely cultured in serum‐free Neurobasal medium (21103049, Gibco/Thermo Fisher Scientific) supplemented with 2% B‐27 Neural Mixture (17504044, Gibco/Thermo Fisher Scientific), 10 ng/mL epidermal growth factor (EGF) (C046, Novoprotein), and 10 ng/mL basic fibroblast growth factor (FGF) (C029, Novoprotein). Cell differentiation was induced by culturing the cells in medium supplemented with 10% (v/v) fetal bovine serum (FBS) (A5256701, Gibco/Thermo Fisher Scientific) for 48 h. All the cells were cultured in a humidified chamber containing 5% CO_2_. The conversion of suspended cells to adherent culture was induced by seeding the cells on poly‐L‐ornithine‐coated plates.

### 4.5 Transient Transfection and Lentiviral Infection

For transient transfection, the siRNA was delivered using 4 µL of Lipofectamine 3000 (Thermo Fisher Scientific) and 5 µL of siRNA (the final concentration was adjusted as needed), while the plasmid DNA was transfected at a 1 µg:1 µL ratio per well in 6‐well plates. All transfections were performed according to the manufacturer's instructions for Lipofectamine 3000, with a standard incubation duration of 48 h. For lentiviral infection, cells were seeded and, after 24 h, infected with lentivirus in the presence of 5 µg/mL polybrene. After 72 h, stable cell lines were established by selection with 2 µg/mL puromycin for 2 weeks. The sequences of the siRNAs targeting KAT7 were shown in Table S3.

### 4.6 Real‐Time qPCR (qRT‐PCR)

Total RNA was isolated from cells using an RNA‐Quick Purification Kit (ES Science, Shanghai, China) with on‐column DNase digestion to eliminate genomic DNA contamination. Complementary DNA (cDNA) was synthesized from 1 µg of total RNA using the ReverTra Ace qPCR RT Kit (Toyobo, Osaka, Japan). Quantitative PCR (qPCR) was performed with TB Green Master Mix on a Roche LightCycler 480 system (Roche, Indianapolis, IN, USA). Gene expression levels were calculated using the ΔΔCt method and normalized to that of β‐actin (ACTB), which served as the internal reference gene. The sequences of the primers used for qPCR were shown in Table S3.

### 4.7 Western Blotting

Cells were lysed in RIPA buffer (Beyotime) supplemented with protease/phosphatase inhibitors and centrifuged (12,000 rpm, 10 min, 4°C), after which the supernatants were collected. The protein concentration was measured using a BCA kit (Beyotime). The samples were mixed with loading buffer (Beyotime, 4:1), denatured (95°C, 5 min), and separated by SDS‒PAGE. The proteins were transferred to PVDF membranes, blocked with 5% skim milk/TBS‐T (1 h), and incubated overnight at 4°C with primary antibodies. After washes with TBS‐T, the membranes were probed with an HRP‐conjugated secondary antibody (1:5000, 1 h) and visualized using ECL (Merck Millipore). The antibodies used were as follows: anti‐MYST2 (KAT7) from Proteintech; anti‐SOX2 from Cell Signaling Technology; anti‐acetyl‐histone H3 (Lys14) from Cell Signaling Technology and ABclonal; anti‐histone H3 from Cell Signaling Technology; anti‐acetyl‐histone H4‐K5 from ABclonal; anti‐acetyl‐histone H4‐K8 from ABclonal; anti‐acetyl‐histone H4‐K12 from ABclonal; anti‐histone H4 from Cell Signaling Technology; anti‐Nestin from Proteintech; anti‐Olig2 from Proteintech; anti‐RAC2 from Abcam; anti‐PAK1/2/3 from ABclonal; anti‐phospho‐PAK1/2/3‐S144/S141/S139 from ABclonal; and anti‐β‐actin from Proteintech.

### 4.8 Immunofluorescence (IF)

Cells were seeded on coverslips and then fixed with 4% paraformaldehyde. Immunofluorescence staining was conducted to determine the subcellular localization of the KAT7 and SOX2 proteins with the following antibodies: Anti‐MYST2 (Proteintech) and anti‐SOX2 (Cell Signaling Technology). A confocal microscope (Leica TCS SP8; Wetzlar, Germany) was used to obtain images of GBM cell immunofluorescence images

### 4.9 Cell Proliferation Assay

Cell proliferation kinetics were systematically evaluated using a CCK‐8 assay (Beyotime Biotechnology, Shanghai, China) through sequential measurements at 0 h (baseline), 24, 48, 72, and 96 h, with patient‐derived glioblastoma stem cells (GBM#P3 at 5 × 10^3^ cells/well and GBM#BG5 at 1 × 10^4^ cells/well) cultured in 96‐well plates under standardized conditions. Following the experimental treatments, 10 µL of CCK‐8 reagent was added to each well and incubated at 37°C for 30 min to allow complete formazan formation. Afterward, we measured the absorbance at 450 nm.

### 4.10 EdU Assay

The cells were labeled with 10 µm EdU for 4 h, fixed with 4% paraformaldehyde, and permeabilized with 0.5% Triton X‐100. Click chemistry reactions were performed using Alexa Fluor‐conjugated azides, followed by DAPI nuclear staining. Fluorescence images were captured, and EdU‐positive cells were quantified using ImageJ.

### 4.11 Apoptosis Assay

Cells (4 × 10^5^) were plated in 6‐well plates and transfected with the siRNA the next day. Cells were washed with PBS, resuspended in staining buffer, and incubated with Annexin V‐FITC and PI (BD Biosciences) for 15 min. All the cells were examined using a BD Biosciences C6 flow cytometer, and the results were processed using FlowJo software (V10, BD Biosciences).

### 4.12 Limiting Dilution and Sphere Formation Assays

For in vitro limiting dilution assays, GSCs were plated into one well of low‐attachment 96‐well plates at the given densities (2, 4, 8, 16, 32, 64, and 128 cells), with 10 replicates for each density. The presence and quantity of tumor spheres in each well were determined 7 days later.

For tumor sphere formation assays, glioblastoma stem cells (GSCs) were plated at a density of 1000 cells/mL in 6‐well ultralow adhesion plates (Corning Inc.) or general plates (Corning Inc.). Following a 10‐day incubation period in Neurobasal medium supplemented with growth factors, tumor sphere development was quantitatively assessed using an inverted phase‐contrast microscope (Nikon Eclipse Ti).

### 4.13 Cell Invasion Assay

Tumor spheroids (GBM#P3, 3 × 10^3^ cells/spheroid; GBM#BG5, 5 × 10^3^ cells/spheroid) were embedded in an invasion matrix (Trevigen, Gaithersburg, MD, USA) and photographed at different times for statistical analysis. The sphere diameter was chosen as the starting point for quantification.

### 4.14 Animal Studies

Five male nude mice (4 weeks old) were randomly assigned to each group and intracranially injected with luciferase‐expressing GBM#P3 cells (3 × 10^5^ cells suspended in 10 µL PBS) into the frontal lobe. The burr hole was positioned 1 mm anterior and 2 mm lateral to the anterior fontanelle, with an injection depth of 2.5 mm. Tumor growth was monitored using bioluminescence imaging (IVIS Spectrum In Vivo Imaging System, PerkinElmer, Hopkinton, MA, USA). For the treatment group, WM‐3835 (5 mg/kg) or saline was administered via intravenous tail vein injection starting at day 7 post‐implantation. Mice were euthanized upon exhibiting signs of persistent discomfort. Brains were harvested and fixed in 4% paraformaldehyde for subsequent hematoxylin‐eosin (HE) staining and immunohistochemical (IHC) analysis.

### 4.15 Immunohistochemistry (IHC)

Tissue sections (4–5 µm) were deparaffinized in xylene and rehydrated through an ethanol gradient. Antigen retrieval was performed using citrate or EDTA buffer (pH 6.0–9.0) with heating. Endogenous peroxidase activity was blocked with 3% H_2_O_2_, and nonspecific binding was blocked with 5% BSA. Primary antibodies were applied, and the sections were incubated overnight at 4°C. After the sections were washed, HRP‐conjugated secondary antibodies were added and incubated for 1 h at room temperature. The signal was developed using DAB substrate, followed by counterstaining with hematoxylin. The sections were dehydrated, cleared, and mounted for imaging under a bright‐field microscope. The primary antibodies used were rabbit anti‐MYST (KAT7) (13751‐1‐AP, 1:50; Proteintech, Wuhan, China), rabbit anti‐Ki67 (GB111499, 1:400; Servicebio, Wuhan, China), and rabbit anti‐RAC2 (ab2244, 1:50; Abcam, Waltham, MA, USA).

### 4.16 RNA‐Seq

TRIzol was used to extract total RNA, followed by DNase treatment to remove genomic DNA. PolyA‐tailed mRNA was enriched using beads, fragmented, and reverse‐transcribed into cDNA. For strand‐specific libraries, dUTP was incorporated during second‐strand synthesis, followed by UDG digestion to preserve directional information. After end repair and adapter ligation, the libraries (300±50 bp) were PCR‐amplified and sequenced. The raw data were subjected to QC, alignment, and transcript quantification. Differential expression and functional enrichment analyses were performed to identify key pathways.

### 4.17 Chromatin Immunoprecipitation (ChIP) Assays and ChIP‐Seq

First, 1% formaldehyde was used to crosslink the cells, followed by quenching with 125 mm glycine. An EZ‐Magna ChIP A/G Chromatin Immunoprecipitation Kit (MERCK, 17–10086) was used for this assay. The cells were lysed, and the chromatin was sheared into 100–500 bp fragments by sonication. For immunoprecipitation, the chromatin was incubated overnight at 4°C with target‐specific antibodies and Protein A/G magnetic beads and then sequentially washed with low/high‐salt buffers. Crosslinks were reversed by heating at 65°C for 6 h. For ChIP‐seq, the DNA was subjected to end repair (20°C for 30 min → 65°C for 30 min), adapter ligation (diluted adapters, 20°C for 15 min), USER enzyme treatment (37°C for 15 min) to excise uracil bases, and PCR amplification (8–10 cycles of 98°C for 10 s, 65°C for 30 s, and 72°C for 30 s). Libraries were selected based on size (300 ± 50 bp) using AMPure XP beads, quantified via Qubit, and validated with high‐sensitivity DNA chips. Finally, paired‐end sequencing (PE150) was performed on the Illumina NovaSeq 6000 platform. For ChIP‒qPCR, DNA was purified by phenol‒chloroform extraction, and target enrichment was quantified using SYBR Green qPCR with primers designed to span peak regions.

### 4.18 ATP Assays

Cells in culture medium were plated in opaque‐walled multiwell plates, with optional background control wells. After 100 µl of CellTiter‐Glo 2.0 Cell Viability Assay (Promega) was added to 100 µl of medium containing the test compounds, the cells were incubated with the compounds. The plate was mixed for 2 min on an orbital shaker to lyse the cells, followed by a 10‐min incubation at room temperature to stabilize the luminescent signal. Luminescence was then measured, with the signal intensity directly proportional to the ATP content.

### 4.19 Modified Seahorse XF Mitochondrial Stress Test

GBM#P3 and BG5 cells were seeded onto Seahorse XF24 V7 PS cell culture microplates (Agilent Technologies). Following treatment with the KAT7 inhibitor, the cells were washed twice with Seahorse XF DMEM (pH 7.4) supplemented with 10 mm glucose, 2 mm glutamine, and 1 mm pyruvate and then equilibrated in 500 µL of fresh medium for 60 min in a non‐CO_2_ incubator at 37°C. The mitochondrial stress test was performed using a Seahorse XFe24 Analyzer with sequential injections of oligomycin (1.5 µm), FCCP (1 µm), and rotenone/antimycin A (500 nm). OCR measurements were recorded every 8 min (3 cycles per injection). The data were normalized to total protein content (µg/well) quantified after the assay using a BCA assay.

### 4.20 TCA Cycle Targeted Flux

GBM#P3 cells were cultured with 10 mm D‐glucose‐13C6 (MCE) for 24 h, and metabolites were extracted from the cells using methanol and derivatized with 3‐nitrophenylhydrazine. The metabolites were analyzed using a Jaspar HPLC system coupled to a Sciex 4500 MD mass spectrometer. The chromatographic conditions were as follows: column, Phenomenex Kinetex C18 (100 × 2.1 mm, 2.6 µm); mobile phase A, 0.1% formic acid in water; and mobile phase B, 0.1% formic acid in acetonitrile.

### 4.21 Surface Plasmon Resonance (SPR)

The recombinant human KAT7 (HBO1/MYST2) protein (Active) (ab268698) was obtained from Abcam. SPR analysis was conducted using the SensiQ Pioneer platform (ForteBio; Fremont, CA, USA). The HisCap biosensor chip (ForteBio) was initially activated with 1 mm NiCl2, followed by immobilization of 50 µg/mL His‐KAT7 protein. Binding assays were performed with the small molecule inhibitor WM‐3835 (500 µm). The interaction signals were recorded as response units (RU) and normalized to control samples. Data analysis was performed using Qdat software (ForteBio), and binding curves were subsequently generated.

## Author Contributions

**J.L. and Y.S**. contributed equally to this work. **B.H., M.H., and X.L**. designed the study. **J.L., Y.S., Y.Z., G.M., JZ.W., L.W., and F.Z**. performed the experiments and acquired the data. **J.L., Y.S., Z.Z., J.W., and Y.H**. analyzed the data. **J.L. and Y.S**. drafted the manuscript. **B.H., M.H., Z.J., and X.L**. reviewed and edited the manuscript.

## Funding

This study was funded by the Key R&D Program of Shandong Province (2025CXPT177), the Natural Science Foundation of China (82504154, 82573856), the China Postdoctoral Science Foundation (2024M761832), the Shandong Provincial Natural Science Foundation (ZR2025QC1768, ZR2022MH137), the Special Foundation for Taishan Young Scholars (tsqn202211041), the Department of Science & Technology of Shandong Province (SYS202202) and the Qilu Young Scholar Program of Shandong University, China and the Special Foundation for Taishan Scholars (tshw201502056).

## Ethics Statement

This study adhered to the ethical standards for human research outlined in the Declaration of Helsinki and was approved by the Qilu Hospital, Shandong University Ethics Committee. All animal experiments complied with institutional ethical guidelines and received approval from the university's Institutional Animal Care and Use Committee.

## Consent

All authors agreed to submit the article for publication.

## Conflicts of Interest

The authors declare no conflicts of interest.

## Supporting information




**Supporting File 1**: advs77551‐sup‐0001‐SuppMat.docx.

**Supporting File 2**:: advs77551‐sup‐0002‐SuppMat.tif.

**Supporting File 3**: advs77551‐sup‐0003‐TableS1.xlsx.

**Supporting File 4**: advs77551‐sup‐0004‐TableS2.xlsx.

**Supporting File 5**: advs77551‐sup‐0005‐TableS3.docx.

## Data Availability

The data that support the findings of this study are openly available in Gene Expression Omnibus at https://www.ncbi.nlm.nih.gov/geo/, reference numbers GSE328130, GSE328308.
