# Supplementary material for: Targeting the KAT7/H3K14ac/RAC2 Axis Mediates OXPHOS to Promote Stemness Maintenance and Malignancy in Glioblastoma
Source: Adv Sci (Weinh). 2026 Sep 8:e77551. Online ahead of print. doi: 10.1002/advs.77551 (PMC13554370; doi:10.1002/advs.77551)
Supplement: Supplementary file 1 [file ADVS-9999-e77551-s004.docx]

**Supplement Table 3: sequences of the siRNAs and primers**

**sequences of the siRNAs**

| Gene | sequences |
| --- | --- |
| siKAT7-1 | 5′-GCUACAACUUCAAUAUGAAGU-3’ |
| siKAT7-2 | 5′-GGCAGUAGUUCAGAUGGAACC-3’ |
| siSOX2-1 | 5′-CCAAGACGCUCAUGAAGAAGG-3’ |
| siSOX2-2 | 5′-GGGACAUGAUCAGCAUGUAUC-3’ |
| siRAC2 | 5′-CACCGUGUUUGACAACUAUUC-3’ |
| siNC | 5′-UUCUCCGAACGUGUCACGUTT-3’ |

**primers for qRT-PCR and ChIP**

| Gene | Forward primer | Reverse primer |
| --- | --- | --- |
| KAT7 | 5′-AGAATATGCACGGCTGGGAC-3′ | 5′-GCGGAGTATCGTTTGGCTCT-3′ |
| RAC2 (H3K14ac) | 5′-CTTGTTTCTTTTTCCCCCTTCAAGA-3′ | 5′-TACGGTGTATACATGCATTGGAA-3′ |
| ACTB | 5′-GAAGAGCTACGAGCTGCCTGA-3′ | 5′-CAGACAGCACTGTGTTGGCG-3′ |
